# Visual hallucinations in Lewy body disease: pathophysiological insights from phenomenology

**DOI:** 10.1007/s00415-022-10983-6

**Published:** 2022-01-31

**Authors:** Fabrizia D’Antonio, Maddalena Boccia, Antonella Di Vita, Antonio Suppa, Andrea Fabbrini, Marco Canevelli, Francesca Caramia, Marco Fiorelli, Cecilia Guariglia, Stefano Ferracuti, Carlo de Lena, Dag Aarsland, Dominic ffytche

**Affiliations:** 1 Department of Human Neuroscience, “Sapienza” University of Rome, Viale dell’Università, 30 00165 Rome, Italy; 2 Department of Psychology, Sapienza University of Rome, Rome, Italy; 3 Cognitive and Motor Rehabilitation Unit, IRCSS Fondazione Santa Lucia, Rome, Italy; 4 IRCCS Neuromed Institute, Pozzilli, IS Italy; 5 IRCCS San Raffaele, Rome, Italy; 6 Institute of Psychiatry, Psychology and Neuroscience, King’s College London, London, UK

**Keywords:** Visual hallucination, Lewy body dementia, Parkinson dementia, Neuroimaging, Misperceptions

## Abstract

Visual hallucinations (VH) in Lewy body disease (LBD) have a heterogenous phenomenology classified into minor phenomena (MVH) and complex hallucinations (CVH). Mechanisms underpinning VH and their temporal aspects are largely unknown. According to the hodotopic model, we investigated whether changes in distinct cognitive domains and neural networks in the hallucination trait underpin temporal aspects of MVH and CVH in the hallucination state. 35 LBD patients with VH underwent a complete neuropsychological evaluation and resting-state fMRI. North-East-Visual-Hallucinations-Interview was used to assess their typical VH content, duration, and frequency. We found that MVH was not associated with cognitive impairment, while CVH was associated with impairments in visuoperceptual processes, attention and visual abstract reasoning. In seed-to-seed functional connectivity (FC) analysis we identified functional couplings associated with MVH and CVH temporal severity (duration x frequency), duration and frequency. MVH severity was negatively associated with FC between early visual areas (EVA) and ventral-visual-stream regions, and negatively associated with FC between brainstem and EVA, which may be linked to LBD brainstem neuropathology. CVH duration was positively associated with FC between ventral-visual stream and salience network (SN). CVH frequency was negatively associated with FC between DMN and SN. Functional alterations in distinct visual and attentional networks and their dynamic interaction in trait LBD hallucinators are linked to both the phenomenology of state content and its temporal characteristics. Within a network, VH frequency and duration may be linked to different types of functional alterations: increased connectivity leading to sustained activity prolonging VH (duration) and decreased connectivity increasing dysregulated, spontaneous activity (frequency). These findings support the hodotopic hypothesis of VH and may reflect a link between VH phenomenology, LBD neuropathological progression and the involvement of specific neurotransmitter systems.

**Supplementary Information:**

The online version contains supplementary material available at 10.1007/s00415-022-10983-6.

## Introduction

The mechanisms underpinning visual hallucinations in Lewy Body Disease (LBD) are largely unknown. LBD represents a spectrum including Parkinson’s disease (PD), Parkinson’s disease dementia (PDD), and dementia with Lewy bodies (DLB) [1–3], which share the same neuropathological marker, namely the intracellular inclusion of alpha-synuclein (Lewy bodies (LB)), as well as clinical features such as visual hallucinations (VH). Many theories have been proposed to explain the brain mechanisms underlying these phenomena. In LBD, models focusing on complex VH contents highlight alterations in cognitive and perceptual mechanisms. The Activation-Input-Modulation (AIM) Model, hypothesizes the need of the three factors for the hallucination’s onset. An activator factor which could be the level of arousal or an environmental factor such as a low ambient stimulation, an input factor which relies on the gating balance between internal stimulus generation and exterior perceptions, and a modulation factor which is the modulatory effects of the neurotransmitters [4]. According to the Perception and Attention Deficit (PAD) model, VH have their roots in the co-occurrence of visuoperceptual impairment and attentional domain alterations, which results in an unbalance between bottom-up and top-down processing [5]. Furthermore, the Attention and Control Model by Shine and colleagues [6, 7] posits that VH are due to reduced engagement of the dorsal attention network (DAN), associated with a greater engagement of the ventral attention network (VAN), and the intrusion of the default mode network (DMN), resulting in greater salience devoted to the stimuli and intrusion of internal thought respectively. Shine and colleagues provided evidence for this model using fMRI during visual stimulation: PD patients with VH showed decreased activation in the frontal eye field and parietal lobe (part of the DAN), which was associated with increased activity in the DMN and VAN when ambiguous stimuli were presented [8]. Overall, AIM, PAD and Attention and Control Models point out a key role of attention. Previous studies showing attentional impairment in PDD patients [9] and both attentional and verbal learning impairments in PD patients [10] have confirmed this idea. Also in DLB patients, a visual attention deficit has been shown to be closely linked to VH [11, 12]. Beyond LBD, the deafferentation-hyperexcitability model of VH posits that altered excitability in the visual associative cortices due to deafferentation is the main mechanism underpinning the simple and complex VH of eye and visual pathway disease, as has been demonstrated in the context of Charles Bonnet syndrome [13, 14]

The hodotopic framework provides a functional anatomical context for such models and the mechanisms underlying VH. Thus, it integrates accounts emphasizing the topological role of functionally specialized brain regions with accounts highlighting the role of large-scale brain networks. It posits that spontaneous increases in activity in specialized visual cortices at the time of a hallucination (the hallucination state) that defines the content of a VH, occurs in the context of longer-term network alterations (the hallucination trait) which underlie a predisposition towards VH [15]. Only a few studies that mainly focused on the role of the DMN have explored the functional alterations associated with VH in LBD. In brief, these studies found that DMN activity was higher in PD with VH and reduced in PD without VH [16]. Additionally, PD patients with VH showed higher functional couplings between specific nodes of the DMN, namely the precuneus, posterior cingulate cortex, and right middle frontal regions, than PD without VH [17].

The phenomenology of VH in LBD is heterogenous and comprises several types of minor and complex hallucinations. Minor phenomena (MVH) include *illusions*, *presence* hallucinations (i.e., the feeling that someone is close by or has just left the room), and *passage* hallucinations (i.e. the vision of a shadow of something or someone passing fast in the periphery of eye field) [14, 18]. Complex visual hallucinations (CVH) in the LBD spectrum, consist of well-formed visions of insects, people (familiar or unfamiliar, alive or dead), inanimate objects, animals, animated figures (often children), normal-size figures, or miniature people [19]. More broadly, phenomenology can also be considered to include temporal aspects of VH such as how long each hallucination lasts and how frequently the hallucinations occur. According to the hodotopic approach, VH phenomenology can help reveal both localized brain regions and brain networks involved in VH. For example, in the PD spectrum VH phenomenology may track disease progression according to Braak stage [20]. When LBs affect the brain stem, MVH occur due to dysfunction in networks connecting subcortical regions such as the brainstem to cortical visual areas, particularly the dorsal visual stream. At later disease stages when LBs progress toward the forebrain and limbic regions, CVH appear. CVH may be either the result of direct involvement of ventral-visual stream regions or the result of cholinergic deafferentation from subcortical regions to ventral-visual stream regions [14].

Despite the high prevalence and associated poor outcomes of CVH and MVH in LBD [14], there are currently no evidence-based medications to treat them or an understanding of the mechanisms underlying how frequently they occur or their duration. A better understanding of the underlying pathophysiology of these temporal phenomenological aspects would potentially point to novel treatment approaches. The aim of the present study was to identify VH neural correlates in LBD from a hodotopic perspective, identifying VH ‘trait’ network alterations that mark a predisposition to specific contents (whether CVH or MVH) and temporal features (their duration or frequency). These trait changes need not be directly related to the ‘state’ activity underlying the hallucinations themselves, but influence this activity when it occurs. To this end we investigated cognitive profiles and resting-state functional connectivity couplings associated with MVH and CVH temporal phenomenology. In terms of cognitive profile we hypothesized that MVH are associated with visuospatial impairment due to dorsal visual stream involvement, whereas CVH are associated with visuoperceptual impairment due to ventral-visual stream involvement. In terms of networks, based on ffytche’s VH model [14], we hypothesized functional alterations between the brainstem and visual cortices and within the dorsal visual stream to underpin MVH, and functional alterations within the ventral-visual stream to underpin CVH. Given the lack of previous evidence and exploratory nature of our analysis of temporal aspects of hallucination duration and frequency we had no specific hypotheses as to what specific aspects of connectivity would relate to temporal phenomenology.

## Materials and methods

### Participants

We included both DLB and PDD participants consistent with the view that both are part of the LBD spectrum [3, 21]. Thirty-nine LBD patients (21 with DLB and 18 with PDD) with a history of VH (as defined below) were recruited from the Department of Human Neuroscience of Sapienza University Hospital of Rome. Patients were considered trait hallucinators if they had experienced repeated hallucinations during the course of their illness. The majority of these patients had ongoing VH at the time of recruitment or the preceding months. We set an arbitrary cut off of having hallucinations in the preceding five years and a minimum of two separate hallucination episodes to define the hallucination trait but, in practice, the average time since the last hallucination was 2.6 months for CVH and 4.8 months for MVH with a maximum of 36 months (see results). DLB diagnosis was made according to the consensus criteria for probable DLB [22], whereas PDD diagnosis was made according to the Movement Disorder Society Clinical Diagnostic Criteria for Parkinson’s Disease [23]. Exclusion criteria for all participants were: MRI contraindications, a previous history of alcohol or substance abuse, significant neurological or psychiatric history, severe ocular diseases (e.g. glaucoma, macular degeneration), epilepsy, other forms of dementia, focal brain lesions on brain imaging, or the presence of other severe or unstable medical illnesses. All participants or their caregivers gave their written informed consent for the study, which was approved by the local ethics committee.

### Clinical and neuropsychiatric assessment

All patients underwent a neurological examination including the Unified Parkinson’s Disease Rating Scale (UPDRS) part III [24] and a structured clinical interview with caregivers to assess the presence of at least two of the core DLB clinical features: parkinsonism (i.e. hypokinesia, rest tremor, postural instability, rigidity), REM sleep behavioral disorders, cognitive fluctuations, and VH. All participants had normal or corrected to normal vision and a gross deficit in color perception was excluded by clinical screening in which patients were asked to indicate a specific color among many different color targets. Current treatment with levodopa, dopamine agonists (DA), antipsychotics, and acetylcholinesterase inhibitors (AchEI), e.g. rivastigmine, was also recorded. Levodopa and DA dosage were converted using the levodopa equivalent scale [25], while antipsychotic drug dosage was converted using the chlorpromazine scale [26]. Neuropsychiatric assessment was performed using the Neuropsychiatric Inventory (NPI) [27, 28].

### Visual hallucination assessment

A modified version of the North-East Visual Hallucinations Interview (NEVHI) [29] was administered to all patients and their caregivers by experienced clinicians/researchers. The NEVHI is a semi-structured interview that assesses in detail the phenomenology of VH including its temporal aspects and time of last VH and its emotional, social, and behavioral impact. The modified version included separate sections for *simple*, *presence*, *illusion*, *complex*, and *passage* hallucinations and *other visual phenomena* with questions about their frequency and time duration. The duration question asks “Approximately how long do these experiences usually last?” and the possible answers are “seconds”, “minutes”, “hours”, or “continuous”, scored respectively from 1 to 4. The frequency question asks “How often do they usually occur?” and the possible answers are: “less than every few months”, “every few months”, “every few weeks”, “every few days”, “every few hours”, “every few minutes”, “every few seconds”, or “continuously”, scored respectively from 1 to 8. The following temporal severity scores were derived:

MVH severity – the sum of duration X frequency for *illusions*, *presence* and *passage* and *other* (possible score 4 – 128).

MVH duration—the sum of duration for *illusions*, *presence* and *passage* and *other* (possible score 4 – 16).

MVH frequency—the sum of frequency for *illusions*, *presence* and *passage* and *other* (possible score 4 – 32).

CVH severity—duration X frequency for *complex* (possible score 1 – 32).

CVH duration—duration *complex* (possible score 1 – 4).

CVH frequency—frequency *complex* (possible score 1 – 8).

Simple hallucinations are considered distinct from MVH or CVH [30] and were only reported by three of the participants (see results) so were not included in the scale.

### Neuropsychological assessment

All patients underwent the Mini-Mental State Examination (MMSE) [31] to measure global cognitive decline, as well as the Activities of Daily Living (ADL) and Instrumental Activities of Daily Living (IADL) [32] tests to assess functional impairment. Patients underwent an extensive neuropsychological evaluation including: (1) Rey’s Auditory Verbal Learning Test [33], Corsi Block-Tapping Test [34], Digit Span [35], Babcock Story Recall Test [34], and Rey-Osterrieth Complex Figure Test [33] for the memory domain; (2) the Visual Search Test [34] and Trail-Making Test [36] for the attentional domain; (3) the Clock Drawing Test [37] and Copy of the Rey-Osterrieth Complex Figure Test [33] for visuo-constructional skill assessment; (4) Semantic and Phonemic Verbal Fluency Test and Boston Naming Test for language [38]; (5) Raven’s Colored Progressive Matrices (RCPM) [39] for abstract reasoning ability and (6) the Frontal Assessment Battery [40] for executive functions. On a different day, patients also underwent the Visual Object and Space Perception battery (VOSP) [41], and the “Length Match Task”, “Size Match Task”, “Orientation Match Task”; “Position of Gap Match Task” subtests of the Birmingham Object Recognition Battery (BORB) [42] and the Benton lines test [43], to assess early visual processing abilities and complex visuoperceptual functions. Visuoperceptual and visuospatial accuracy indices were computed for each participant using VOSP subtest scores as follows: the visuoperceptual index corresponded with the mean of the proportions of correct answers on the Incomplete Letters, Silhouettes, Object Decision, and Progressive Silhouettes; the visuospatial index corresponded to the mean of the proportions of correct answers on the Dot Counting, Position Discrimination, Number Location and Cube Analysis. Since, a higher score corresponds to a worse performance on the silhouette subtest, while a higher score indicates better performance on all other VOSP subtests, we inverted the silhouette score before computing the index in order to homogenize the correspondence between score and performance. A BORB accuracy index was also computed as the mean of the proportion of correct answers on the administered subtests.

### Statistical analysis

Statistical analyses were performed using SPSS (v. 25). First, spearman correlation coefficients were computed to test the association between MVH and CVH severity scores and demographics (age and education), concomitant medications (levodopa, DA, AchEI, antipsychotics), and parkinsonism severity (UPDRS). Spearman correlation coefficients were used to test the association between MVH and CVH severity, duration, and frequency scores and neuropsychological test scores with significance threshold of *p* < 0.05 Bonferroni corrected for 150 multiple comparisons (equivalent to uncorrected *p* < 0.0003).

### Image acquisition and analyses

MRI scans were collected on a Siemens Magnetom Verio 3-Tesla scanner. Functional T2*-weighted images were collected using a gradient echo sequence to measure the blood oxygen level-dependent (BOLD) contrast over the whole brain. During resting-state fMRI scans the patients were taking their routine medication and asked to lay at rest with eyes closed and not to fall asleep. Head movements were minimized with a mild restraint and cushioning. Functional MRI images were acquired in the interleaved mode for the entire cortex using BOLD contrast imaging (200 fMRI scans, 50 slices, in-plane resolution = 3 × 3 mm, slice thickness = 3 mm, repetition time (TR) = 3 s, echo time (TE) = 31 ms). Resting-state data were processed using the CONN toolbox for functional connectivity analysis (v. 16a) [44] (http://www.nitrc.org/projects/conn) running on Statistical Parametric Mapping 12 (SPM12) software (http://www.fil.ion.ucl.ac.uk/spm). We used the standard pre-processing pipeline in the CONN toolbox. In brief, after removal of the initial four scans, the functional images were resampled to a voxel size of 2 × 2 × 2 mm^3^, realigned and unwarped; time series were interpolated to correct for slice-timing distortions. Structural images were segmented in gray matter, white matter (WM), and cerebrospinal fluid (CSF) for successive use during removal of temporal confounding factors and normalized to MNI space. ART-based scrubbing [45] for detection of functional outliers was also applied (data showing framewise displacement greater than 0.9 mm or with a z-normalized signal change over the whole brain greater than 5 were discarded along with the preceding and the following two timepoints). According to the default settings, seed-to-seed connectivity analysis was performed on unsmoothed data aggregated across all voxels within each seed ROI. Temporal confounding factors (i.e. time-courses of WM and CSF BOLD signals, a linear trend, and the six motion parameters derived from the previous realignment procedure) were removed from the BOLD time series of functional data, regressing them out at each voxel. A band-pass filter (0.008–0.09 Hz) was then applied to resulting residual time series.

In this study a model-based/hypothesis-driven analysis approach was used to test intrinsic functional coupling between seed regions we expected to be involved in VH, based on previous studies and theoretical models (Supplementary Table 1). Thus, we included as seeds early visual areas, i.e. Occipital pole (OP) and Cuneal cortex, regions of the ventral-visual stream, i.e. temporo occipital fusiform cortex (TOFusC) and occipital fusiform gyrus (OFusG), Lingual gyrus (LG), inferior division of lateral occipital cortex (iLOC), temporo occipital part of inferior temporal gyrus (toITG), anterior and posterior division of parahippocampal gyrus (aPaHC, pPaHC), regions of the dorsal visual stream, i.e. superior division of lateral occipital cortex (sLOC), temporo occipital part of middle temporal gyrus (toMTG), regions of the salience network, i.e., anterior and posterior division of supramarginal gyrus (aSMG, pSMG), and regions part of the default mode network, i.e. angular gyrus (AG), posterior division of cingulate gyrus (PC), Precuneus and finally brain stem based on ffytche’s hypothesis [14]. Since we were interested in the intrahemispheric connectivity of these nodes, and given that resting-state signal fluctuations in cortical regions tend to be highly positively correlated between homologous regions across the two hemispheres, we analyzed each hemisphere separately, as follows. Using multiple regression models, we performed a seed-to-seed functional connectivity (FC) analysis to disclose possible specific nodes whose functional coupling was associated with the: (i) severity, (ii) duration, and (iii) frequency of MVH (controlling for CVH and MMSE) and CVH (controlling for MVH and MMSE) for the right and the left hemisphere. Cluster-level inference was adopted to control for family-wise error rate, as implemented in Conn (20.b; CONN toolbox www.nitrc.org/projects/conn, RRID:SCR_009550). In brief, rather than focusing on individual connections between all possible pairs of ROIs, cluster-level inference focuses on groups of nearby or related connections sharing similar effects or results. Here we used default hierarchical clustering method and parametric multivariate statistics (Functional Network Connectivity; [46], with a cluster threshold of p < 0.05 cluster-level p-FDR corrected (MVPA omnibus test) and a connection threshold of *p* < 0.05 p-uncorrected.

## Results

Data from 35 out of 39 patients were analyzed, since 2 patients were excluded due to a visual impairment and 2 were excluded from fMRI analysis due to movement artifacts. The final sample included 19 DLB patients and 16 PDD patients. The sample included 23 males (65.7%). Mean age was 76.7 (± 6.5) years, mean time from disease onset was 4.3 (± 2.8) years, mean time since last CVH was 2.6 (± 6.8) months, mean time since last MVH was 4.8 (± 6.2) months and the mean MMSE score was 20.7 (± 5.8), indicating a mild global cognitive impairment on average. All the participants were right-handed. Demographic and clinical data are shown in Table 1.

**Table 1:** Demographics and clinical characteristics of LBD patients (DLB *n* = 19, PDD *n* = 16)

Variable	Mean (± SD)
Age (years)	76.7 (± 6.5)
Education (years)	9 (± 3.9)
Gender (Male, number and %)	23 (65.7%)
Time from disease onset (years)	4.3 (± 2.8)
MMSE	20.7 (± 5.8)
UPDRS	23.3 (± 11.7)
NPI total score	28.9 (± 16.1)
NEVHI total score	11.7 (± 10.9)
NEVHI Severity of minor hallucinations	4.7 (± 8.2)
NEVHI Severity of complex hallucinations	6.9 (± 4.9)
NEVHI duration of minor hallucinations	1.4 (± 1.8)
NEVHI duration of complex hallucinations	3.5 (± 1.9)
NEVHI frequency of minor hallucinations	2.6 (± 3.02)
NEVHI frequency of complex hallucinations	3.5 (± 1.7)
Time from last CVH episode (months)	2.6 (± 6.8)
Time from last MVH episode (months)	4.8 (± 6.2)
AcheI (number of patients in therapy and %)	19 (54.2%)
AcheI dose	5.4 (± 4.3)
Levodopa (number of patients in therapy and %)	19 (± 54.2%)
Levodopa equivalent dose	364.1 (± 378.8)
Antipsychotics (number of patients in therapy and %)	14 (40%)
Antipsychotics (Chlorpromazine equivalent dose)	36 (± 57.3)

**Fig. 1:**
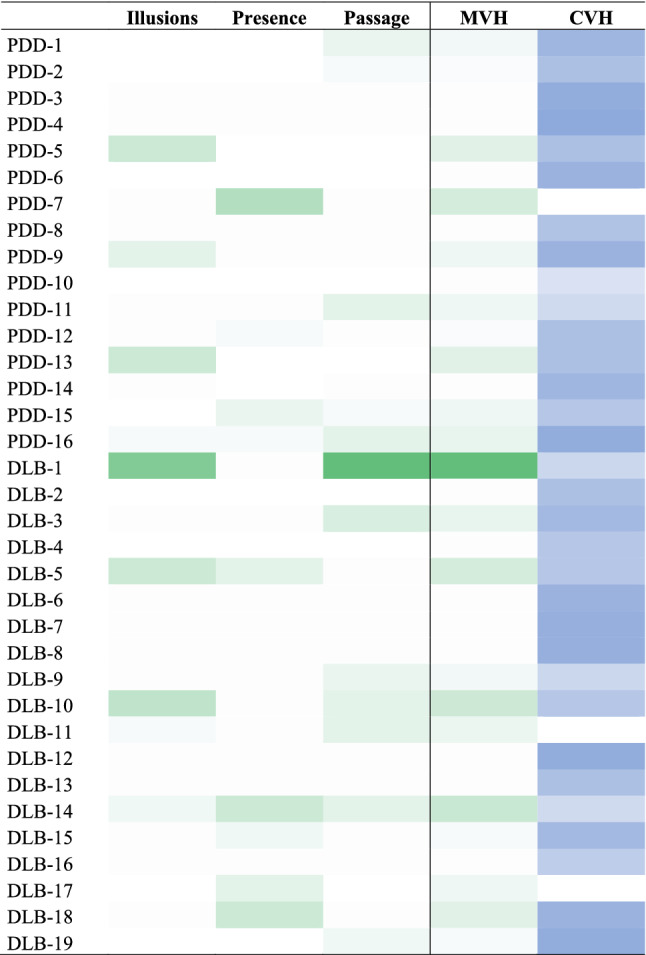
Colored patches of visual phenomena in LBD patients. Minor visual hallucinations, *MVH* light green-to-dark green; complex visual hallucinations, *CVH* light blue-to-dark blue represent the severity of symptoms reported by each patient for each phenomenon

Nine patients had *illusions*, 9 patients had *presence* hallucinations, 12 had *passage* hallucinations, 32 had *complex* hallucinations (3 patients had minor phenomena unassociated with CVH (Fig. 1). Only three patients reported simple visual hallucinations. None of the patients experienced VH during resting-state fMRI scanning or reported falling asleep.

No significant correlations were found between MVH and CVH severity scores, and age, education, time from disease onset, concomitant medications, or UPDRS score.

### Neuropsychological results

No significant correlations were found between cognitive test scores and MVH severity (the mean test scores are shown in Supplementary Table 2 and Spearman’s correlation coefficients are reported in Table 2). We explored the possibility that the lack of significant correlation might be a statistical artifact related to 0 values (i.e. patients who did not report MVH). We therefore repeated the analysis without participants with 0 values and also looked for a difference in cognitive score between patients with and without MVH. Neither analysis suggested an association between MVH and cognitive test score, other than at trend significance for TMT-A and TMT-B (see Supplementary Table 3 and Supplementary Table 4). In contrast, a negative correlation was detected between CVH severity and MMSE (*r* =  – 0.568, p_corr_ < 0.05), VOSP visuoperceptual index (*r* =  – 0.625, *p*_corr_ < 0.05), RCPM (*r* =  – 0.581, *p*_corr_ < 0.05) and visual search scores (*r* =  – 0.622, *p*_corr_ < 0.05) at trend significance for VOSP spatial index (*r* =  – 0.562, uncorrected *p* = 0.002). CVH duration was negatively associated with RCPM score (*r* =  – 0.577, *p*_corr_ < 0.05) and at trend significance for VOSP visuoperceptual index (*r* =  – 0.535, uncorrected *p* = 0.003), VOSP spatial index (*r* =  – 0.559, uncorrected *p* = 0.002), visual search scores (*r* =  – 0.496, uncorrected *p* = 0.003) and MMSE (*r* =  – 0.471, uncorrected *p* = 0.004).

**Table 2:** Spearman correlation coefficients (r) between Minor (MVH) and complex visual hallucinations (CVH) scores and neuropsychological test scores and significance (*p*)

*Test*	Value	MVH severity	CVH severity	MVH duration	CVH duration	MVH frequency	CVH frequency
*MMSE*	*r*	0.045	– 0.568*	0.069	– 0.471	– 0.010	– 0.450
	*p*	0.797	0.000	0.694	0.004	0.957	0.007
*Benton*	*r*	– 0.073	– 0.357	– 0.017	– 0.314	– 0.111	– 0.285
	*p*	0.706	0.057	0.932	0.097	0.565	0.135
*VOSP visuo*	*r*	– 0.070	– 0.625*	– 0.020	– 0.535	– 0.084	– 0.497
	*p*	0.717	0.000	0.917	0.003	0.663	0.006
*VOSP spatial*	*r*	– 0.121	– 0.562	– 0.078	– 0.559	– 0.127	– 0.406
	*p*	0.530	0.002	0.687	0.002	0.513	0.029
*BORB*	*r*	– 0.083	– 0.412	– 0.077	– 0.379	– 0.094	– 0.364
	*p*	0.682	0.033	0.704	0.051	0.641	0.062
*DS*	*r*	– 0.135	– 0.402	– 0.107	– 0.412	– 0.094	– 0.208
	*p*	0.455	0.020	0.555	0.017	0.601	0.246
*CBT*	*r*	0.000	– 0.363	0.040	– 0.316	0.009	– 0.221
	*p*	0.998	0.038	0.825	0.073	0.961	0.216
*RAVL IR*	*r*	0.047	– 0.238	0.067	– 0.286	0.046	– 0.108
	*p*	0.796	0.183	0.711	0.107	0.800	0.549
*RAVL DR*	*r*	0.197	– 0.317	0.240	– 0.361	0.226	– 0.221
	*p*	0.271	0.072	0.178	0.039	0.205	0.217
*Babcock IR*	*r*	0.089	– 0.114	0.128	– 0.121	0.030	– 0.115
	*p*	0.629	0.536	0.484	0.510	0.870	0.530
*Babcock DR*	*r*	– 0.085	– 0.235	– 0.043	– 0.194	– 0.076	– 0.198
	*p*	0.645	0.196	0.815	0.287	0.681	0.277
*RCFT IR*	*r*	0.185	– 0.005	0.235	– 0.013	0.158	– 0.035
	*p*	0.327	0.979	0.211	0.945	0.405	0.856
*RCFT DR*	*r*	0.261	– 0.053	0.311	– 0.244	0.256	0.131
	*p*	0.164	0.781	0.094	0.195	0.171	0.490
*TMT-A*	*r*	– 0.224	0.180	– 0.237	0.211	– -0.155	0.059
	*p*	0.218	0.324	0.192	0.247	0.397	0.750
*TMT-B*	*r*	– 0.376	0.059	– 0.363	0.048	– 0.355	0.044
	*p*	0.064	0.780	0.075	0.818	0.081	0.835
*VS*	*r*	– 0.177	– 0.622*	– 0.155	– 0.496	– 0.210	– 0.558
	*p*	0.323	0.000	0.390	0.003	0.240	0.001
*PVF*	*r*	– 0.052	– 0.350	-0.039	– 0.288	– 0.038	– 0.244
	*p*	0.772	0.046	0.831	0.104	0.835	0.171
*SVF*	*r*	– 0.202	– 0.461	-0.209	– 0.363	– 0.279	– 0.302
	*p*	0.269	0.008	0.252	0.041	0.123	0.093
*BNT*	*r*	0.094	– 0.529	0.137	– 0.496	0.065	– 0.368
	*p*	0.607	0.002	0.453	0.004	0.723	0.039
*CDT FD*	*r*	– 0.049	– 0.508	0.015	– 0.371	– 0.111	– 0.420
	*p*	0.816	0.009	0.942	0.068	0.596	0.037
*CDT ED*	*r*	0.074	– 0.419	0.122	– 0.267	0.034	– 0.351
	*p*	0.725	0.037	0.562	0.197	0.870	0.086
*CDT PD*	*r*	– 0.151	– 0.421	– 0.067	– 0.309	– 0.155	– 0.308
	*p*	0.463	0.032	0.744	0.124	0.450	0.126
*RCFT copy*	*r*	0.086	– 0.210	0.149	– 0.233	0.032	– 0.151
	*p*	0.647	0.257	0.422	0.207	0.864	0.417
*RCPM*	*r*	– 0.172	– 0.581*	– 0.131	– 0.577*	– 0.162	– 0.395
	*p*	0.340	0.000	0.466	0.000	0.367	0.023
*FAB*	*r*	– 0.116	– 0.330	– 0.100	– 0.260	– 0.154	– 0.290
	*p*	0.527	0.065	0.588	0.150	0.399	0.107

Significant correlation coefficients with CVH that survived the Bonferroni’s correction for multiple comparison are marked with an asterisk

Notes: *IR*: immediate recall, *DR* delayed recall, *RAVLT* Rey's auditory verbal learning test, *DS* Digit span, *CBT* Corsi block tapping test, *RCFT* Rey‐Osterrieth complex figure test, *VS* visual search test, *TMT‐A* trail making test part A, *TMT‐B* trail‐making test part B, *PVF* phonemic verbal fluency, *SVF* semantic verbal fluency, *BNT* boston naming test, *CDT* clock drawing test, *FD* free drawing condition, *PD* pre-drawn condition, *ED* examiner-drawn condition, *RCPM*. Raven's colored progressive matrices; *FAB* frontal assessment battery

**Fig. 2:**
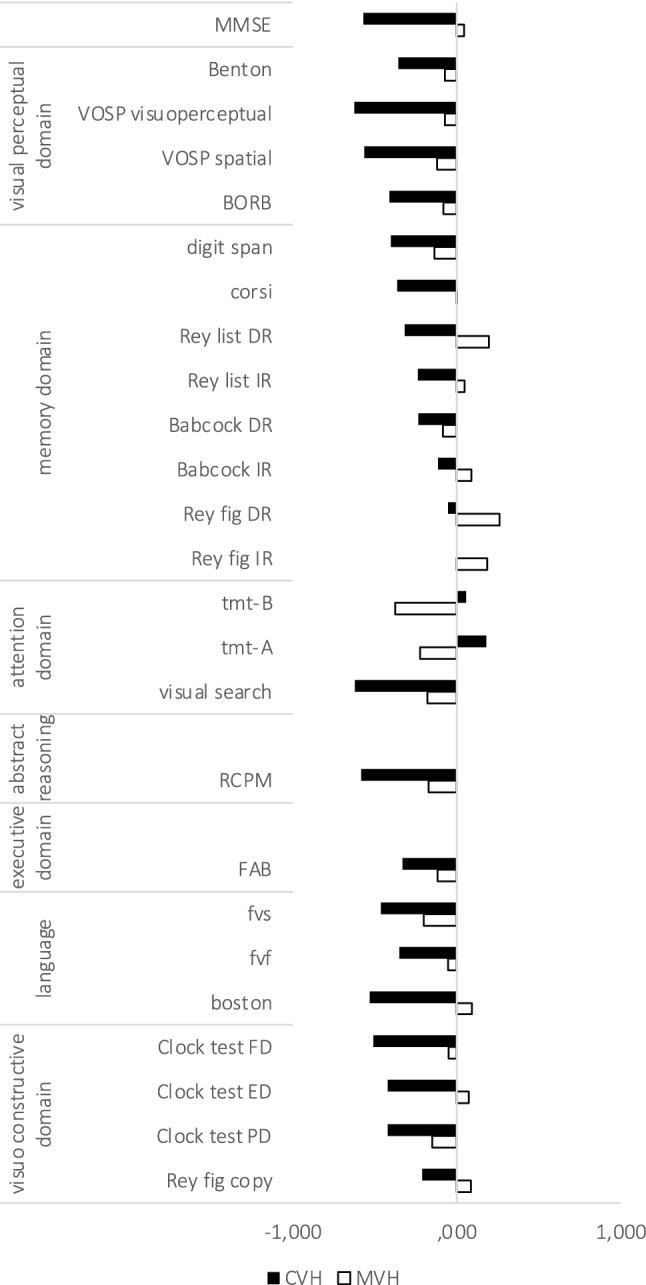
Bars summarize Spearman’s coefficients between minor (MVH) and complex (CVH) severity obtained from NEVHI and neuropsychological data. Abbreviations: *IR* immediate recall, *DR* delayed recall, *TMT‐A* Trail-Making Test part A, *TMT‐B* Trail‐Making Test part B, *RCPM* Raven’s colored progressive matrices, *FAB* Frontal Assessment Battery, *Clock test FD* free drawing condition, *PD* pre-drawn condition, *ED* examiner-drawn condition, *PVF* Phonemic Verbal Fluency, *SVF* Semantic Verbal Fluency

The correlation trends between complex and minor hallucinations and neuropsychological domains are shown in Fig. 2. Spearman’s correlation coefficients are reported in Table 2 along with significance.

## Neuroimaging results

### MVH seed-to-seed results

**Fig. 3:**
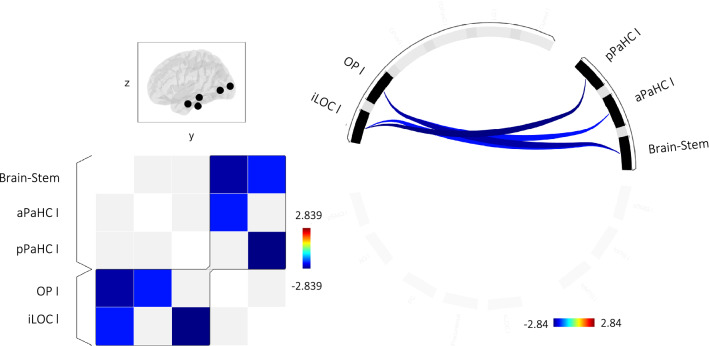
Network of minor visual phenomena. Groups of related ROIs has been defined using hierarchical clustering procedure. Significant connections were identified using a cluster threshold of *p* < 0.05 cluster-level *p*-FDR corrected (MVPA omnibus test) and a connection threshold of *p* < 0.05 p-uncorrected. Regions showing suprathreshold functional couplings associated with minor visual phenomena (i.e. severity) are depicted in light blue-to blue patches (on the left panel) and line (on the right panel). Notes: *l* left, *aPaHC* anterior parahippocampal cortex, *pPaHC *posterior parahippocampal cortex, *OP* occipital pole, *iLOC* inferior lateral occipital complex

Figure 3 shows the network of related ROIs sharing similar functional metrics defined using the hierarchical clustering procedure, and, in this network, the regions showing suprathreshold functional couplings associated with minor visual phenomena. Specifically, the multiple regression models revealed that seed-to-seed FC between brain stem and OP and iLOC was negatively associated with MVH severity in the left hemishere. Also, FC between posterior parahippocampal gyrus and iLOC and between anterior parahippocampal gyrus and OP were negatively associated with MVH severity in the left hemisphere. No significant effects were detected in the right hemisphere. Moreover, MVH duration and frequency were not associated with FC alterations neither in the right or left hemisphere. Statistics are reported in Table 3.

**Table 3:** Significant associations between minor visual hallucinations and regional functional couplings

	Seed		T_(31)_	p-unc	p-FDR
*Right hemisphere*					
Severity					
		No significant suprathreshold cluster			
Duration					
		No significant suprathreshold cluster			
Frequency					
		No significant suprathreshold cluster			
*Left hemisphere*					
Severity					
		F(2,30) = 8.76		0.001	0.015
	Brain stem	Occipital pole l	− 2.65	0.012	
	Posterior parahippocampal gyrus l	Inferior division lateral occipital cortex l	– 2.84	0.007	
	Brain stem	Inferior division lateral occipital cortex l	– 2.05	0.049	
	Anterior parahippocampal gyrus l	Occipital pole l	– 2.04	0.049	
Duration					
		No significant suprathreshold cluster			
Severity					
		No significant suprathreshold cluster			

Notes on region labels: l = left hemisphere

### CVH seed-to-seed results

**Fig. 4:**
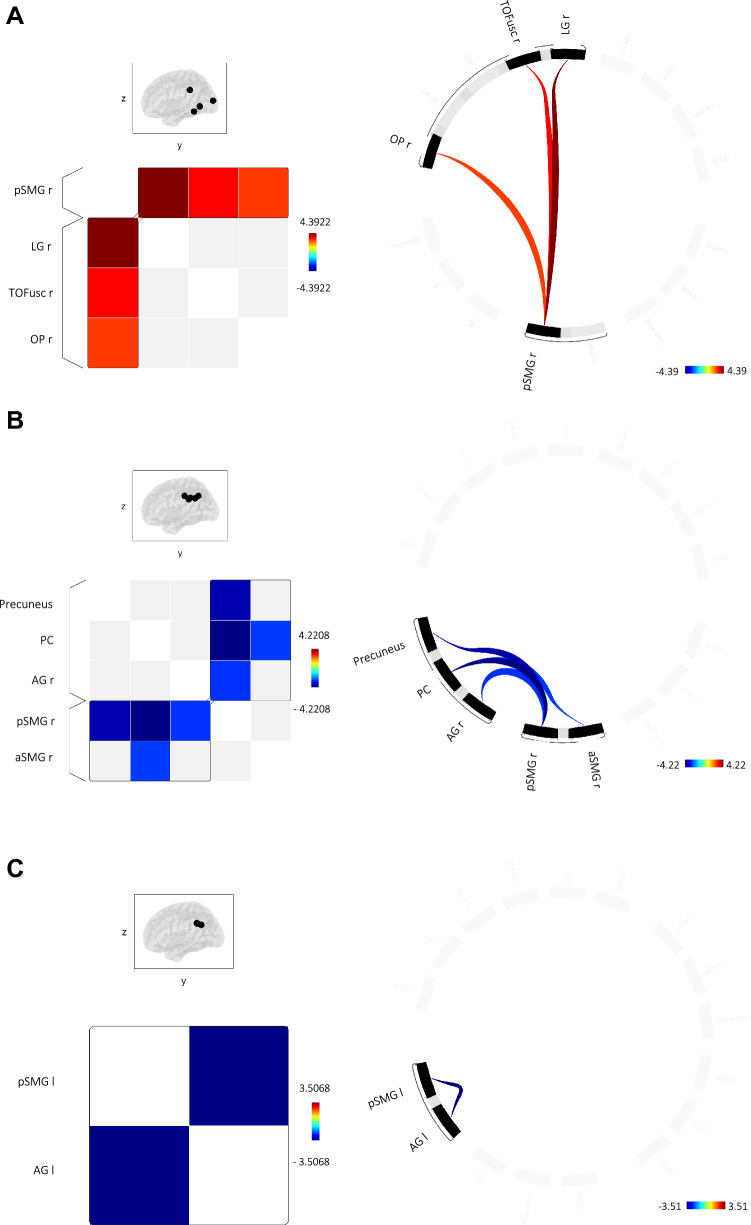
Network of complex visual hallucinations. Groups of related ROIs has been defined using hierarchical clustering procedure. Significant connections were identified using a cluster threshold of *p* < 0.05 cluster-level p-FDR corrected (MVPA omnibus test) and a connection threshold of *p* < 0.05 p-uncorrected. Regions showing suprathreshold functional couplings associated with complex visual hallucinations are depicted in light blue-to blue patches (on the left) and line (on the right) when a negative relation has been found; otherwise, positive association with complex visual hallucinations are depicted in yellow-to-red patches and lines. Panels display the resting-state functional couplings whose strengths showed a significant association with duration of complex visual hallucinations in the right hemisphere (**A**) and that with frequency in the right (**B**) and left hemisphere (**C**). Notes: *l*  left, *r* right, *OP* occipital pole, *PC* posterior cingulate cortex, *AG* angular gyrus, *pSMG* posterior supramarginal gyrus, *aSMG* anterior supramarginal gyrus, *LG* lingual gyrus, *TOFusC* temporoccipital fusiform cortex

Figure 4 shows the networks of related ROIs sharing similar functional metrics defined using the hierarchical clustering procedure, and the regions showing suprathreshold functional couplings associated with CVH duration and frequency. Thus, CVH severity was not associated with FC alterations, neither in the right or left hemisphere. However, FC between pSMG and LG, TOFus and OP was positively associated with CVH duration in right hemisphere. Also, FC between Precuneus and right aSMG and pSMG was negatively associated with CVH frequency, in the right hemisphere; FC between right PC and right pSMG and between right AG and right pSMG was negatively associated with CVH frequency in the right hemisphere, as well. Finally, FC between AG and SMG was negatively associated with CVH frequency in the left hemisphere. No significant associations with CVH duration were found in the left hemisphere. Statistics are reported in Table 4.

**Table 4:** Significant associations between duration and frequency of complex visual hallucinations and regional functional couplings

	Seed		T_(31)_	p-unc	p-FDR
*Right hemisphere*					
Severity					
		No significant suprathreshold cluster			
Duration					
		F(2,30) = 7.53		0.00224	0.03366
	Posterior division supramarginal gyrus r	Lingual gyrus r	4.39	0.00012	
	Posterior division supramarginal gyrus r	Temporoccipital fusiform cortex r	3.34	0.0021	
	Posterior division supramarginal gyrus r	Occipital pole r	2.79	0.00883	
Frequency					
		F(2,30) = 6.96		0.00329	0.04940
	Posterior division cingulate gyrus r	Posterior division supramarginal gyrus r	– 4.22	0.00019	
	Precuneus	Posterior division supramarginal gyrus r	– 3.88	0.00050	
	Precuneus	Anterior division supramarginal gyrus r	– 2.72	0.01054	
	Angular gyrus r	Posterior division supramarginal gyrus r	– 2.80	0.00869	
*Left Hemisphere*					
Severity					
		No significant suprathreshold cluster			
Duration					
		No significant suprathreshold cluster			
Frequency					
		F(1,31) = 12.30		0.00140	0.02110
	Angular gyrus l	Posterior division supramarginal gyrus l	– 3.51	0.00140	

Notes on region labels: l  left hemisphere, *r*  right hemisphere

## Discussion

The present study investigated alterations in cognitive functioning and resting-state functional couplings in patients with LBD susceptible to VH (hallucination trait). Neuropsychological and functional coupling alterations were identified when patients were not hallucinating that correlated with temporal aspects of the VH experience itself (hallucination state), suggesting VH trait alterations influenced VH state activity. By examining minor and complex VH separately, we tested the hypothesis that temporal aspects of the two classes of phenomena are underpinned by distinct neuropsychological profiles and functional networks. In support of this hypothesis, we found MVH were not associated with cognitive impairment while CVH were associated with abstract reasoning, visuoperceptual, and attentional alterations and that functional alterations across visual and related networks differed for MVH and CVH. For easiness of exposition the discussion will be divided into subheadings, concerning the cognitive profile we found to be associated with temporal aspects of VH in LBD, and functional networks associated with MVH and CVH.

### Cognitive profile associated with VH in LBD

When cognitive score correlations were analyzed separately for MVH and CVH, no significant associations were found between impairment in any cognitive domain and MVH. These findings suggest that MVH are not associated with impairments in cognitive and perceptual processes measured by the wide-ranging tests used in this study. It may be an as yet unspecified visual dysfunction produces the “erroneous” perceptions of MVH without cognitive impairment as assessed by the tests employed. We do not think the lack of an association between MVH and cognitive measures reflects the bias related to zero values as confirmed by the correlations performed excluding participants without MVH (see Supplementary Table 3). This result is consistent with findings of the only previous study that explored the neuropsychological profile associated with MVH, reporting no relation between MVH and cognitive impairments [47]. In contrast CVH were associated with global cognitive impairment and deficits in visuoperceptual processing, visual attention, and abstract reasoning related to visual patterns and textures. These results are consistent with the hypothesis that impairment in visuoperceptual function related to the ventral-visual stream, “what pathway” involved in object recognition [5] is a prerequisite for complex hallucinations. The overall temporal severity of CVH was related to impairments in visual perception coupled with impaired reality monitoring through attentional, reasoning and global cognition deficits. Of note these associations seemed to be primarily driven by the duration measure (correlation with non-verbal reasoning abilities [Raven’s Colored Progressive Matrices, a test in which subjects are presented sequences of visual patterns and textures and asked to choose the next in the sequence] and, at trend, global cognition) and not the frequency measure (see below for discussion of duration versus frequency). For CVH, the results support the validity of the neuropsychological Perceptual and Attention model [5] which states that impairment in visuoperceptual processing may lead to a failure in the bottom-up process, which cannot be compensated by an effective top-down mechanism due to cognitive impairment.

Our results are in line with previous studies that have reported an association between VH in LBD and impairments in visuoperceptual function [48, 49], visual recognition [50], visual attention [11, 12], attention [9, 10]. Previous studies also found an impairment in executive functions to be associated with VH [12, 47, 51–53]. In this study we did not found this association, although we found a correlation between CVH severity and RCPM, that may be also considered a measure of executive function [54–57]. However, previous studies did not investigate possible associations between neuropsychological functions and VH content or the temporal phenomenology of duration or frequency, which might account for the difference in findings.

Overall our cognitive findings are consistent with the hypothesis we set out to test that the profile of deficits for MVH is distinct from that of CVH. The associations found between temporal aspects of CVH phenomenology and specific cognitive functions in trait hallucinators suggest these functions influence activity underlying the hallucinations state for CVH. Conversely, the lack of associations found for MVH suggest the same functions do not influence activity underlying the hallucination state for MVH.

### Minor hallucinations and FC

We found FC alterations in trait hallucinators associated with temporal aspects of MVH, the implication being that these network changes influenced activity underlying the MVH state. MVH severity was associated in the left hemisphere with reduced FC between the primary visual cortex and parahippocampal regions as well as with a decreased FC between parahippocampal regions and inferior lateral occipital cortex part of the visual stream. Moreover, we found that a decreased FC between the brain stem and primary visual area and the inferior lateral occipital cortex.

The MVH scale is a composite of *illusion*, *passage* and *presence* subscales and it is likely different nodes are responsible to different phenomenological aspects of MVH content.

The FC alteration with the lateral occipital cortex, a region specifically involved in the object recognition, i.e. iLOC [58], may account for *illusions*, whereas the altered FC with parahippocampal region may account for *passage* interpreting this result in light of the parahippocampal role in visuospatial processing. It is known that parahippocampus specifically responds to place and building recognition (i.e. parahippocampal place area), however additional hypotheses have been proposed for its role [59]. Levi et al. proposed that the ventral stream is organized along a center-periphery gradient and that parahippocampus might process objects that are typically in the periphery of the visual field [60].

Also, the parahippacampal region is in close proximity to the newly described area prostriata that specifically processes information from the periphery of the visual field and shows a preferential response for fast motion visual stimuli [61].

An altered connectivity with this region may account for the passage hallucination that typically occur as unformed figures passing fast in the periphery of the eye field. Finally, the decreased connectivity between brainstem and primary visual area and object recognition area supports ffytche’s hypothesis that alteration of the subcortical control on visuoperceptual process contributes to minor phenomena [14]. Conversely, we did not find alterations involving dorsal visual stream areas. It is possible that these alterations occur during the hallucination state, whereas alterations in spatial recognition process represent the context (trait) within which minor phenomena, such as illusions, passage and presence hallucinations, occur.

No associations were found between FC and MVH duration and frequency considered separately. The reasons for this are unclear but these phenomena usually occur earlier than CVH during the LBD disease course [14] and are more transient and less temporally defined. The questionnaire measures might not be sensitive enough to show associations with duration or frequency when considered separately but are able to do so when combined in the temporal severity measure.

FC connectivity alterations associated with MVH were found just in the left hemisphere. The reason for this is unclear and may relate to the individual symptom components of the MVH. It might be, for example, dysfunction in left hemisphere networks with a relative specialization for processing local features results in certain types of illusion, contrasting with right hemisphere networks specialized for processing global features [62]. The area prostriata has a left hemisphere bias which may relate to the passage hallucination component of the MVH scale.

Overall our results are consistent with previous structural MRI studies that have shown cortical atrophy in PD patients with minor VH in regions of the visual stream and subcortical atrophy of the superior colliculus [63] and peduncolo-pontine nucleus, which may affect cholinergic transmission from the brainstem to the cortex [64]. Our results are also consistent with previous functional studies of MVH in PD that reported diffuse hypometabolism in upper brainstem and posterior cortical regions and distributed network alterations within visual associative cortex [65, 66].The pattern of FC alterations associated with MVH support ffytche’s hypothesis that MVH are underpinned by altered activation from the brainstem to visual areas [14].

### Complex hallucinations and FC

We found FC alterations in trait hallucinators associated with temporal aspects of CVH, the implication being that these network changes influenced activity underlying the CVH state.

CVH duration was associated in the right hemisphere with increased FC between primary visual area, fusiform cortex and the lingual gyrus in the ventral-visual stream and the supramarginal gyrus, part of the SN. These findings suggest that a possible mechanism underlying CVH is dysregulation among the ventral-visual stream areas and salience network with VH resulting from increased salience attributed to the internally-generated perceptual contents. Shine’s model also interpreted the increased engagement of the VAN as a condition of greater salience attributed to the stimuli [6]. Functional connectivity alterations associated with CVH duration were found just in right hemisphere. This result can be interpreted considering the ventral attention network contribution to CVH. Indeed, VAN has a right hemisphere predominance and its tracts are more represented in the right hemisphere [67]. A previous neuroimaging study investigating VH in DLB patients found that microstructural and functional alterations involving attention networks in the right hemisphere contribute to VH, sustaining that VH are associated with an abnormal functioning of the VAN in the right hemisphere [68]. Overall these results suggest that altered bottom-up processes related to areas in VAN and SN are implicated for CVH occurrence.

Conversely, frequency-related FC revealed that alterations in the DMN and SN are linked to CVH in both hemispheres. The angular gyrus and posterior cingulate gyrus are both part of the DMN and were found to be less connected with the supramarginal gyrus of the SN. The role of the angular gyrus in the DMN seems to be linked to the manipulation of conceptual knowledge and mental representations when the mind wanders at rest [69]. Our results suggest that the reduced salience devoted to these internal thoughts is associated with CVH frequency. Contrary to previous studies [8, 16, 17], and to Shine’s Attention and Control model, we found a decreased FC in the DMN and SN. A possible explanation is that the Attention and Control model has been tested by means of a paradigm based on ambiguous perceptual stimuli more related to misperception illusions than CVH, so may be more relevant to MVH than CVH. Also, our study relates specifically to temporal aspects of VH rather than susceptibly to VH in general as tested in the Shine paradigm. Our results are in line with findings from previous functional studies reporting altered recruitment of the ventral-visual stream and a large-scale multi-network derangement associated with VH in DLB [70], as well as diffuse desynchronization of the lingual gyrus [71], consistent with posterior/occipital changes typical of DLB [72–74].

Overall the FC results are consistent with the neuropsychological findings of impairments in visuoperceptual abilities, visual attention and visual abstract reasoning associated with CVH. Thus, FC alterations involving ventral-visual stream may explain the visuoperceptual alterations and the decreased FC between DMN and SN may be linked to the alterations in attention and reasoning abilities (linked to the angular gyrus) associated with CVH.

For CVH, duration and frequency considered individually were associated with FC but not the combined severity measure, the opposite of what was found for MVH. This might be explained by differences in the construction of the two scales. CVH relates to just one type of complex phenomenon reported by the patient as the most severe while the MVH severity score is the sum of each individual minor phenomenon severity and captures the range of minor phenomena in addition to temporal aspects.

### Network connectivity and hallucination duration and frequency

This is the first study to explore temporal aspects of hallucinations and we had no specific a priori hypotheses to test. In terms of the hodotopic framework, one might anticipate the duration of hallucinations (lasting seconds, minutes, hours etc.) to be related to persistent, sustained activity in the network underlying the symptoms. One possibility is that this type of driving activity is a consequence of hyper-connection/increased coupling in the network, as reflected in a positive correlation with duration, for example between the SN and ventral-visual stream for CVH. In contrast, the frequency of hallucinations (occurring every month, week, day etc.) might be related to instability within the network either as the result of disconnection / reduced coupling as reflected in a negative correlation with frequency, for example between DMN and SN for CVH. Although speculative, these observations suggest duration and frequency may relate to different aspects of network dysfunction and provide new hypotheses to test in future studies.

### General discussion

The present findings shed light on the mechanisms underpinning VH in LBD. VH may be due to functional alterations in visual networks, with VH phenomenology being defined by the specific brain regions and networks involved. The involvement of specific areas may reflect a link with the neuropathological progression of LBD spread or involvement of specific neurotransmitter systems in the disease process. Visual network involvement may represent the “primum movens” but it is not sufficient for VH onset or temporal phenomenological aspects such as their duration and frequency. These aspects of phenomenology seem to depend on the interaction between visual networks and those of the DMN and SN and alterations of FC that lead to changes on the balance of sustained and unstable, ‘spontaneous’ or ‘autonomous’ activity in networks.

### Clinical applications and future directions

Investigating VH according to their phenomenology both in terms of content and temporal characteristics, helps clarify the mechanisms underpinning VH, detecting the areas, networks and network dysfunctions involved. This approach may have significant translational potential in clinical practice. Differences in phenomenology may translate to differences in the underlying neurotransmitter systems involved with implications for treatment [75]. For example, CVH frequent in DLB, may be related to cholinergic dysfunctions from ascending systems, mainly affecting limbic regions and the ventral-visual stream [76, 77]. MVH may be instead considered part of a syndrome also including metamorphopsia typically occurring in conditions linked to the serotonergic system such as hallucinogen persisting perception disorder [78], MDMA [79] and 5-HT2 antagonism [80]. Understanding the neurotransmitter signature of the hallucinations in LBD according to content could help develop effective treatments for specific VH subtypes. Another implication of the phenomenological differences identified is that duration and frequency of VH may be associated with distinct functional alterations, suggesting different types of treatment may be needed to target different aspects of temporal phenomenology.

### Caveats and Limitations

A limitation of the present study is the indirect nature of the inferences drawn from trait to state hallucinations. While it seems reasonable to infer that associations between trait changes and state phenomenology imply a shared mechanism for both, without a better understanding of the activity occurring at the time of MVH and CVH in LBD, we cannot be certain that networks exhibiting FC alterations in trait hallucinators or the cognitive domains implicated are directly related to the hallucination state. In our cohort we did not investigate the role of the retina in VH occurrence. Since LB retinal burden could also contribute to VH, future studies will clarify how retinal alterations are associated with functional network changes in LBD. Moreover, in this study the patients were not assessed for visual contrast sensitivity, although they were screened for color discrimination deficits.

Another limitation is that we cannot definitively establish the role of specific MVH and CVH content (faces or figures for example). Also it was not possible investigate the FC alterations associated with each MVH. Future studies that include LBD patients experiencing only one type of phenomenon could help disentangle this issue. Future studies should also investigate and compare VH with specific phenomenology in different neurodegenerative disorders, also analyzing PD, PDD and DLB separately, in order to clarify whether the findings are generally applicable to neurodegenerative disease or specific to Lewy body dementias.

## Conclusions

The findings of the present study suggest that VH phenomenology, broadly defined including content and temporal aspects of VH, is an important consideration for studies of VH mechanism. Exploring VH from a phenomenological point of view helps identify different brain regions, networks and network dysfunctions involved. In his *phenomenologie de la perception* (1945), Merlau-Ponty claimed that it is not possible to differentiate the experiencing subject from the experience itself [81]. Restating this in neuroscientific terms it may be that what we perceive or misperceive (i.e. a VH) is closely linked to the specialized brain areas involved in elaborating that specific experience. The hodotopic approach to phenomenology with its focus on functional anatomy and networks may be the model that best accommodates this conceptualization. Future studies will test whether this approach can be considered valid for understanding VH in different pathological contexts.

## Supplementary Information

Below is the link to the electronic supplementary material.

Supplementary file1 (DOC 96 KB)

Supplementary file2 (DOCX 17 KB)

Supplementary file3 (DOCX 22 KB)

Supplementary file4 (DOCX 18 KB)

## Data Availability

The data that support the findings of this study are available from the corresponding author, upon reasonable request.

## Abbreviations

AchEI: Acetylcholinesterase inhibitors
AG: Angular gyrus
AIM: Activation-input-modulation
BNT: Boston naming test
CDT: Clock drawing test
aPaHC: Anterior division of parahippocampal gyrus
aSMG: Division of supramarginal gyrus
CBT: Corsi block tapping test
CVH: Complex visual hallucinations
DA: Dopamine agonists
DAN: Dorsal attention network
DLB: Dementia with Lewy bodies
DMN: Default mode network
DS: Digit span
EVA: Early visual areas
FAB: Frontal assessment battery
FC: Functional connectivity
iLOC: Inferior division of lateral occipital cortex
LB: Lewy bodies
LBD: Lewy body disease
LG: Lingual gyrus
MMSE: Mini mental state examination
MVH: Minor visual hallucinations
NEVHI: North-east visual hallucinations interview
NPI: Neuropsychiatric inventory
OFusG: Occipital fusiform gyrus
OP: Occipital pole
PAD: Perception and attention deficit
PC: Posterior division of cingulate gyrus
PD: Parkinson’s disease
PDD: Parkinson’s disease dementia
PVF: Phonemic verbal fluency
pPaHC: Posterior division of parahippocampal gyrus
pSMG: Posterior division of supramarginal gyrus
RAVLT: Rey's auditory verbal learning test
RCPM: Raven's colored progressive matrices
RCFT: Rey‐osterrieth complex figure test
sLOC: Superior division of lateral occipital cortex
SN: Salience network
SVF: Semantic verbal fluency
TMT‐A: Trail making test part A
TMT‐B: Trail‐making test part B
TOFusC: Temporo occipital fusiform cortex
ToITG: Temporo occipital part of inferior temporal gyrus
toMTG: Temporo occipital part of middle temporal gyrus
VAN: Ventral attention network
UPDRS: Unified Parkinson’s Disease Rating Scale
VH: Visual hallucinations
VS: Visual search test
