# Supplementary material for: Visual hallucinations in Lewy body disease: pathophysiological insights from phenomenology
Source: J Neurol. 2022 Jan 31;269(7):3636–52. doi: 10.1007/s00415-022-10983-6 (PMC9217885; doi:10.1007/s00415-022-10983-6)
Supplement: Supplementary file 1 — Supplementary file1 (DOC 96 KB) [file 415_2022_10983_MOESM1_ESM.doc]

| **ROIs** | **Paper** | **Patients** | **MRI method** | **Notes** | **Results** | | **Network** | |
| --- | --- | --- | --- | --- | --- | --- | --- | --- |
| Lingual gyrus (LG) | Ramirez-Ruiz et al. 2007 | 18 PD VH  20 PD NVH  21 HC | VBM |  | Decreased GM in LG in PD VH vs PD NVH and HC | |  | |
|  | Goldman et al.2014 | 25 PD VH  25 NVH | VBM |  | Decreased GM in LG in PD VH vs PD NVH | |  | |
|  | Peraza et al.2014 | 16 DLB  17HC | rsFMRI |  | Reduced FC between LG and calcarine cortex in DLB vs HC | Ventral stream | |  |
|  | Yao et al. 2015 | 12 PD VH  12 PD NVH  14 HC | rsfMRI |  | Lower ALFF in LG in PD VH vs PD NVH |  | |  |
| Fusiform cortex (FusC) | Goldman et al.2014 | 25 PD VH  25 NVH | VBM |  | Decreased GM in FusC in PD VH vs PD NVH | |  | |
|  | Yao et al. 2015 | 12 PD VH  12 PD NVH  14 HC | rsfMRI |  | Higher ALFF in FusC in PD VH vs PD NVH | Ventral stream | |  |
|  | Hepp et al. 2017 | 15 PD VH  40 PD NVH  15 HC | rsfMRI |  | Reduced FC in FusC in PD VH vs HC |  | |  |
| Parahippocampal gyrus (PaHC) | Yao et al. 2015 | 12 PD VH  12 PD NVH  14 HC | rsfMRI |  | Higher ALFF in parahippocampus in PD VH vs PD NVH | |  | |
|  | Shine et al. 2015 | 21 PD VH  14 PD NVH | task fMRI | *Acquistion during Bistable Percept Paradigm* | During misperceptions decreased activity in the DAN, increased activity in VAN and DMN. Increased FC between DMN and VIS; decreased FC between DAN and DMN, and VAN in PD VH vs PD NVH | Ventral stream | |  |
| Inferior temporal gyrus (ITG) | Yao et al. 2015 | 12 PD VH  12 PD NVH  14 HC | rsfMRI |  | Increased FC of ITG in PD VH vs HC | | Ventral stream | |
| Middle temporal gyrus (MTG) | Hepp et al. 2017 | 15 PD VH  40 PD NVH  15 HC | rsfMRI |  | Reduced FC in MTG in PD VH vs HC | |  | |
|  | Bejr-kasem et al. 2019 | 18 PD VH  14 NVH | rsfMRI |  | Increased FC between the PCC and MTG in PD VH vs PD NVH | Dorsal stream | |  |
| Lateral occipital cortex (LOC) | Meppelink et al. 2009 | 9 PD VH  14 PD NVH  13 HC | Task fMRI | *Images that gradually pop out of random uniform visual white noise* | Reduced activation of LOC in PD VH vs PD NVH and HC before image recognition | | Ventral/Dorsal stream (inferior/superior LOC) | |
|  | Taylor et al. 2012 | 17 DLB  19 HC | Task fMRI | *A block design with three different conditions: checkerboard, objects, and motion stimuli* | Reduced activation to the motion stimulus in LOC in DLB vs HC |  | |  |
| Supramarignal gyrus (SMG) | Lee et al. 2017 | 10 PD VH  21 PD NVH  17 PDD VH  30 HC | VBM |  | Decreased GM in SMG in PD VH vs PD NVH | | Salience network | |
|  | Bejr-kasem et al. 2019 | 18 PD VH  14 NVH | VBM |  | Decreased GM in SMG in PD VH vs PD NVH |  | |  |
| Angular gyrus (AG) | Nagahama et al. 2010 | 100 DLB  20 HC | SPECT | *Factor analysis: four factors of psychotic symptoms identified in SPECT analyses* | Hypoperfusion bilat. angular gyrus associated with person and presence VH compared to the others factors in DLB | |  | |
|  | Shine et al. 2015 | 21 PD VH  14 PD NVH | task fMRI | *Acquistion during Bistable Percept Paradigm* | During misperceptions decreased activity in the DAN, increased activity in VAN and DMN. Increased FC between DMN and VIS; decreased FC between DAN and DMN, and VAN in PD VH vs PD NVH | | Default mode network | |
| Posterior Cingulate Gyrus (PC) | Goetz et al. 2014 | 1 PD VH | fMRI during VH | *single case* | Increased activation of AC during VH | |  | |
|  | Shine et al. 2015 | 21 PD VH  14 PD NVH | task fMRI | *Acquistion during Bistable Percept Paradigm* | During misperceptions decreased activity in the DAN, increased activity in VAN and DMN. Increased FC between DMN and VIS; decreased FC between DAN and DMN, and VAN in PD VH vs PD NVH | Default mode network | |  |
|  | Yao et al. 2014 | 12 PDVH  12 PD NVH  14 HC | rsfMRI |  | Increased FC in bilateral posterior cingulate gyrus in PD VH vs PD NVH |  | |  |
|  | Bejr-kasem et al. 2019 | 18 PD VH  14 PD NVH | VBM |  | Decreased GM in PC in PD VH vs PD NVH |  | |  |
| Precuneus | Yao et al. 2014 | 12 PDVH  12 PD NVH  14 HC | rsfMRI |  | Increased FC in precuneus in PDVH vs PD NVH | |  | |
|  | Shine et al. 2015 | 21 PD VH  14 PD NVH | task fMRI | *Acquistion during Bistable Percept Paradigm* | During misperceptions decreased activity in the DAN, increased activity in VAN and DMN. Increased FC between DMN and VIS; decreased FC between DAN and DMN, and VAN in PD VH vs PD NVH | | Default mode network | |
|  | Bejr-kasem et al. 2019 | 18 PD VH  14 PD NVH | VBM |  | Decreased GM in Precuneus in PD VH vs PD NVH |  | |  |
| Cuneal cortex | Goldman et al.2014 | 25 PD VH  25 NVH | VBM |  | Decreased GM in Cuneus in PD VH vs PD NVH | |  | |
|  | Yao et al. 2015 | 12 PD VH  12 PD NVH  14 HC | rsfMRI |  | Lower ALFF in Cuneus in PD VH vs PD NVH | Early visual area | |  |
| Occipital pole (OP) | Meppelink et al. 2009 | 9 PD VH  14 PD NVH  13 HC | Task fMRI | *Images that gradually pop out of random uniform visual white noise* | Decreased activation of bilateral OP in PD VH vs PD NVH and HC | |  | |
|  | Yao et al. 2015 | 12 PD VH  12 PD NVH  14 HC | rsfMRI |  | Lower ALFF in OP in PD VH vs PD NVH |  | |  |
|  | Lefebvre et al. 2016 | 18 PD VH  16 PD NVH  17 HC | Task fMRI | *Visual detection task (threshold evaluation)* | Right occipital cortex more activated at visual detection threshold  in PD VH vs PD NVH | Early visual area | |  |
|  | Hepp et al.2017 | 15 PD VH  40 PD NVH  15 HC | rsfMRI |  | Reduced FC in in occipital cortex in PD VH vs HC |  | |  |
| Brain stem | Janzen et al. 2012 | 13 PD VH  13 PDD VH  16 PD NVH  11 DLB | VBM |  | Decreased GM in PPN in PD VH and PDD VH vs PD | |  | |
|  | ffytche et al. 2017 |  |  | *Theoretical model of PD Psychosis* |  |  | |  |

**Supplementary Table 1 –** Reference studies on brain functional and structural findings associated with VH in LBD spectrum. Notes. ALFF= Amplitude of Low Frequency Fluctuations. DAN= Dorsal Attention Network. DLB= Dementia with Lewy body. DMN =Default mode Network. FC= Functional connectivity. GM= grey matter. HC= Healthy controls. NVH= not visual hallucinators. PD= Parkinson’s Disease. PDD= Parkinson Disease Dementia. PPN= Pedunculopontine nucleus. rsfMRI= resting state functional Magnetic resonance imaging. SPECT= Single Photon Emission Computed Tomography. VBM= Voxel Based Morphometry. VAN= Ventral Attention Network. VH= Visual hallucination. VIS= Visual networks
