# Supplementary material for: Visual hallucinations in Lewy body disease: pathophysiological insights from phenomenology
Source: J Neurol. 2022 Jan 31;269(7):3636–52. doi: 10.1007/s00415-022-10983-6 (PMC9217885; doi:10.1007/s00415-022-10983-6)
Supplement: Supplementary file 2 — Supplementary file2 (DOCX 17 KB) [file 415_2022_10983_MOESM2_ESM.docx]

| Variable | Mean (SD) |
| --- | --- |
| MMSE | 20.7 (± 5.8) |
| Benton line | 10.6 (± 8.1) |
| BORB | 0.7 (±0.2) |
| VOSP visuoperceptual | 0.5 (±0.1) |
| VOSP visuopspatial | 0.6 (±0.2) |
| RAVLT immediate recall | 18.7 (±8.7) |
| RAVLT delayed recall | 2.2 (± 2.4) |
| Babcock test immediate recall | 2.4 (± 3.1) |
| Babcock test delayed recall | 2.5 (± 2.5) |
| DS | 4.5 (± 1.6) |
| CBT | 3 (± 1.4) |
| RCFT immediate recall | 2.4 (± 3.1) |
| RCFT delayed recall | 2.2 (± 3.5) |
| VS | 26.6 (± 16.1) |
| TMT-A | 183.6 (± 110.8) |
| TMT-B | 249.6 (± 146.2) |
| PVF | 15.3 (± 10.2) |
| SVF | 19.7 (± 7.2) |
| BNT | 25.4 (±11.3) |
| RCFT copy | 7.3 (± 9) |
| CDT FD | 6.8 (± 5.1) |
| CDT PD | 6.3 (± 4.4) |
| CDT ED | 18.4 (± 11) |
| FAB | 9.2 (± 3.7) |
| RCPM | 13.5 (±9.3) |

**Supplementary Table 2-** Neuropsychological characteristics in the LBD patients.

Notes: IR: immediate recall; DR: delayed recall; RAVLT, Rey's auditory verbal learning test; DS, Digit span; CBT, Corsi block tapping test; ; RCFT, Rey‐Osterrieth complex figure test; VS, visual search test; TMT‐A, Trail Making test part A; TMT‐B, Trail‐Making test part B; PVF, Phonemic Verbal Fluency; SVF, Semantic Verbal Fluency; BNT, Boston Naming test; CDT, Clock Drawing test; FD, free drawing condition; PD, pre drawn condition ; ED, examiner drawn condition; FAB, Frontal Assessment Battery; RCPM, Raven's Coloured Progressive Matrices.
