# Supplementary material for: Visual hallucinations in Lewy body disease: pathophysiological insights from phenomenology
Source: J Neurol. 2022 Jan 31;269(7):3636–52. doi: 10.1007/s00415-022-10983-6 (PMC9217885; doi:10.1007/s00415-022-10983-6)
Supplement: Supplementary file 3 — Supplementary file3 (DOCX 22 KB) [file 415_2022_10983_MOESM3_ESM.docx]

| Test | Value | MVH severity | MVH  duration | MVH  frequency |
| --- | --- | --- | --- | --- |
| MMSE | r | -1.00 | -0.042 | -0.253 |
|  | p | 0.667 | 0.858 | 0.268 |
| Benton | r | -0.306 | -0.150 | -0.430 |
|  | p | 0.249 | 0.580 | 0.097 |
| VOSP visuo | r | -0.388 | -0.252 | -0.425 |
|  | p | 0.137 | 0.347 | 0.101 |
| VOSP spatial | r | -0.343 | -0.206 | -0.363 |
|  | p | 0.194 | 0.445 | 0.167 |
| BORB | r | -0.166 | -0.126 | -0.198 |
|  | p | 0.572 | 0.668 | 0.498 |
| DS | r | -0.069 | -0.005 | 0.027 |
|  | p | 0.773 | 0.982 | 0.909 |
| CBT | r | -0.298 | -0.188 | -0.275 |
|  | p | 0.201 | 0.427 | 0.240 |
| RAVL IR | r | 0.122 | 0.167 | 0.111 |
|  | p | 0.609 | 0.481 | 0.642 |
| RAVL DR | r | -0.179 | -0.055 | -0.109 |
|  | p | 0.451 | 0.819 | 0.647 |
| Babcock IR | r | 0.074 | 0.186 | -0.089 |
|  | p | 0.765 | 0.446 | 0.719 |
| Babcock DR | r | -0.117 | 0.004 | -0.105 |
|  | p | 0.633 | 0.988 | 0.670 |
| RCFT IR | r | -0.100 | 0.046 | -0.193 |
|  | p | 0.703 | 0.860 | 0.459 |
| RCFT DR | r | -0.063 | 0.085 | -0.079 |
|  | p | 0.811 | 0.746 | 0.763 |
| TMT-A | r | -0.534 | -0.574 | -0.358 |
|  | p | 0.018 | 0.010 | 0.132 |
| TMT-B | r | -0.632 | -0.603 | -0.597 |
|  | p | 0.006 | 0.010 | 0.011 |
| VS | r | -0.147 | -0.113 | -0.240 |
|  | p | 0.535 | 0.637 | 0.309 |
| PVF | r | -0.236 | -0.209 | -0.190 |
|  | p | 0.316 | 0.378 | 0.422 |
| SVF | r | -0.198 | -0.223 | -0.413 |
|  | p | 0.417 | 0.359 | 0.079 |
| BNT | r | -0.082 | 0.030 | -0.159 |
|  | p | 0.737 | 0.902 | 0.516 |
| CDT FD | r | 0.074 | 0.271 | -0.085 |
|  | p | 0.794 | 0.328 | 0.763 |
| CDT ED | r | 0.058 | 0.198 | -0.074 |
|  | p | 0.838 | 0.480 | 0.793 |
| CDT PD | r | -0.080 | 0.132 | -0.097 |
|  | p | 0.769 | 0.625 | 0.721 |
| RCFT copy | r | -0.019 | 0.157 | -0.149 |
|  | p | 0.941 | 0.533 | 0.556 |
| RCPM | r | -0.143 | -0.052 | -0.142 |
|  | p | 0.548 | 0.827 | 0.551 |
| FAB | r | 0.210 | 0.272 | 0.099 |
|  | p | 0.389 | 0.261 | 0.686 |

**Supplementary Table 3 –** Spearman correlation coefficients (r) between Minor visual hallucinations (MVH) and neuropsychological test scores and significance (p) excluding patients without MVH,

Notes: IR: immediate recall; DR: delayed recall; RAVLT, Rey's auditory verbal learning test; DS, Digit span; CBT, Corsi block tapping test; ; RCFT, Rey‐Osterrieth complex figure test; VS, visual search test; TMT‐A, Trail Making test part A; TMT‐B, Trail‐Making test part B; PVF, Phonemic Verbal Fluency; SVF, Semantic Verbal Fluency; BNT, Boston Naming test; CDT, Clock Drawing test; FD, free drawing condition; PD, pre drawn condition ; ED, examiner drawn condition; RCPM, Raven's Coloured Progressive Matrices; FAB, Frontal Assessment Battery,
