# Supplementary material for: Visual hallucinations in Lewy body disease: pathophysiological insights from phenomenology
Source: J Neurol. 2022 Jan 31;269(7):3636–52. doi: 10.1007/s00415-022-10983-6 (PMC9217885; doi:10.1007/s00415-022-10983-6)
Supplement: Supplementary file 4 — Supplementary file4 (DOCX 18 KB) [file 415_2022_10983_MOESM4_ESM.docx]

|  | MVH Absence  Mean (SD) | MVH Presence  Mean (SD) | Mann-Withney U | p-value |
| --- | --- | --- | --- | --- |
| MMSE | 19.7 (6.1) | 21.3 (5.6) | 131.5 | 0.60 |
| Benton line | 10.6 (7.6) | 10.7 (8.7) | 99.5 | 0.84 |
| BORB | 0.73 (0.13) | 0.66 (0.25) | 87 | 0.84 |
| VOSP visuoperceptual | 0.53 (0.11) | 0.55 (0.17) | 92.5 | 0.61 |
| VOSP visuopspatial | 0.66 (0.24) | 0.64 (0.27) | 103.5 | 0.98 |
| RAVLT immediate recall | 18.08 (8.1) | 19.1 (9.3) | 128.5 | 0.98 |
| RAVLT delayed recall | 1.46 (2.1) | 2.75 (2.5) | 86 | 0.96 |
| Babcock test immediate recall | 1.97 (2.4) | 2.82 (3.5) | 113.5 | 0.68 |
| Babcock test delayed recall | 2.69 (2.8) | 2.4 (2.3) | 116 | 0.76 |
| DS | 4.62 (1.7) | 4.55 (1.6) | 113 | 0.51 |
| CBT | 2.85 (1.6) | 3.1 (1.3) | 110.5 | 0.45 |
| RCFT immediate recall | 1.5 (2.4) | 3.17 (3.5) | 81 | 0.18 |
| RCFT delayed recall | 0.88 (1.5) | 3.35 (4.2) | 73.5 | 0.08 |
| VS | 29.3 (15.3) | 24.8 (16.7) | 104.5 | 0.34 |
| TMT-A | 186.08 (91.9) | 181.8 (124.5) | 121 | 0.92 |
| TMT-B | 285.63 (173.2) | 232.6 (134.1) | 59 | 0.57 |
| PVF | 14.9 (9.7) | 15.6 (10.7) | 124.5 | 0.83 |
| SVF | 22.3 (7.8) | 19.05 (7.1) | 101 | 0.38 |
| BNT | 22.17 (8.19) | 27.4 (13.1) | 101 | 0.38 |
| RCFT copy | 7.07(8.6) | 9.9 (10.7) | 103.5 | 0.58 |
| CDT FD | 7.00 (4.6) | 6.67 (5.6) | 67.5 | 0.67 |
| CDT PD | 7.2 (4.3) | 5.88 (4.5) | 67 | 0.49 |
| CDT ED | 17.1 (11.2) | 19.4 (11.2) | 68.5 | 0.71 |
| FAB | 10.08 (4.4) | 8.63 (3.2) | 91.5 | 0.21 |
| RCPM | 14.6 (8.1) | 12.8 (10.2) | 109.5 | 0.44 |

**Supplementary Table 4 –** Neuropsychological test score differences between patients with and without MVH.

Notes: IR: immediate recall; DR: delayed recall; RAVLT, Rey's auditory verbal learning test; DS, Digit span; CBT, Corsi block tapping test; ; RCFT, Rey‐Osterrieth complex figure test; VS, visual search test; TMT‐A, Trail Making test part A; TMT‐B, Trail‐Making test part B; PVF, Phonemic Verbal Fluency; SVF, Semantic Verbal Fluency; BNT, Boston Naming test; CDT, Clock Drawing test; FD, free drawing condition; PD, pre drawn condition ; ED, examiner drawn condition; RCPM, Raven's Coloured Progressive Matrices; FAB, Frontal Assessment Battery.
